# Tied up in knots: Untangling substrate recognition by the SPOUT methyltransferases

**DOI:** 10.1016/j.jbc.2022.102393

**Published:** 2022-08-18

**Authors:** Sarah E. Strassler, Isobel E. Bowles, Debayan Dey, Jane E. Jackman, Graeme L. Conn

**Affiliations:** 1 Department of Biochemistry, Emory University School of Medicine, Atlanta, Georgia, USA; 2 Graduate Program in Biochemistry, Cell and Developmental Biology, Graduate Division of Biological and Biomedical Sciences, Emory University, Atlanta, Georgia, USA; 3 Department of Chemistry and Biochemistry, Center for RNA Biology and Ohio State Biochemistry Program, Columbus, Ohio, USA

**Keywords:** SPOUT methyltransferase, RNA methylation, S-adenosyl-L-methionine (SAM), ribosomal RNA (rRNA), transfer RNA (tRNA), phylogenetic analysis, substrate recognition, CTD, C-terminal domain, NTD, N-terminal domain, ML, maximum likelihood, SAM, S-adenosyl-L-methionine, SAH, S-adenosyl-homocysteine, SPOUT, SpoU-TrmD

## Abstract

The SpoU-TrmD (SPOUT) methyltransferase superfamily was designated when structural similarity was identified between the transfer RNA–modifying enzymes TrmH (SpoU) and TrmD. SPOUT methyltransferases are found in all domains of life and predominantly modify transfer RNA or ribosomal RNA substrates, though one instance of an enzyme with a protein substrate has been reported. Modifications placed by SPOUT methyltransferases play diverse roles in regulating cellular processes such as ensuring translational fidelity, altering RNA stability, and conferring bacterial resistance to antibiotics. This large collection of S-adenosyl-L-methionine-dependent methyltransferases is defined by a unique α/β fold with a deep trefoil knot in their catalytic (SPOUT) domain. Herein, we describe current knowledge of SPOUT enzyme structure, domain architecture, and key elements of catalytic function, including S-adenosyl-L-methionine co-substrate binding, beginning with a new sequence alignment that divides the SPOUT methyltransferase superfamily into four major clades. Finally, a major focus of this review will be on our growing understanding of how these diverse enzymes accomplish the molecular feat of specific substrate recognition and modification, as highlighted by recent advances in our knowledge of protein–RNA complex structures and the discovery of the dependence of one SPOUT methyltransferase on metal ion binding for catalysis. Considering the broad biological roles of RNA modifications, developing a deeper understanding of the process of substrate recognition by the SPOUT enzymes will be critical for defining many facets of fundamental RNA biology with implications for human disease.

Methyltransferases are a large group of enzymes that catalyze methyl transfer on diverse substrates to perform one of the most common cellular modifications (1). Methylation is important to gene expression, integrity of macromolecular structure and function, and many facets of small molecule metabolism (2, 3, 4, 5). Over 95% of known methyltransferases use S-adenosyl-L-methionine (SAM) as their co-substrate, generating S-adenosyl-homocysteine (SAH) as a product of the methylation reaction. These SAM-dependent enzymes are categorized into five main classes (I-V) based on their catalytic domain structure, although several additional subgroups have recently been identified, including the radical SAM methyltransferases (1, 6, 7).

The largest group, Class I methyltransferases, are characterized by a structurally conserved Rossmann-like fold with a central topological switch point in the seven β-strand core and a GxG(xG) motif that forms the SAM binding pocket (1). Class II methyltransferases are structurally characterized by a long antiparallel β-sheet surrounded by groups of helices. The active site of these enzymes includes a conserved RxxxGY sequence that binds SAM in an extended conformation at a shallow solvent-exposed groove on the surface of the reaction domain (1, 7, 8). In Class III methyltransferases, SAM binds in a folded conformation at the active site located between two αβα domains consisting of five β-strands and four helices (1, 7, 9). The Class IV SAM-dependent methyltransferase family contains the SpoU-TrmD (SPOUT) enzymes, characterized by a unique α/β fold and a deep trefoil knot in the C-terminal half of the SPOUT methyltransferase catalytic domain (10, 11, 12, 13). Finally, Class V methyltransferases, or the SET-domain proteins, are composed mainly of β-strands and form a knot at their C terminus distinct from that of Class IV and bind SAM in a kinked conformation on the enzyme surface (1, 7).

The SPOUT superfamily was first designated when crystal structures confirmed the structural similarity of several methyltransferases, supporting the previously identified sequence homology between two enzymes, SpoU and TrmD, which catalyze different modifications on transfer RNA (tRNA) substrates (10, 14, 15, 16, 17, 18). SpoU was later renamed TrmH to denote its biochemical function as the eighth tRNA methylation gene identified in bacteria (19). SPOUT methyltransferases predominantly methylate tRNA and ribosomal RNA (rRNA) substrates, though one instance of an enzyme that methylates a protein substrate has been reported (20, 21, 22). RNA-modifying SPOUT methyltransferases perform methylation at two different general locations on RNA nucleotides: some, like TrmH, methylate the ribose 2′-OH, while others, including TrmD, perform nucleobase methylation (17, 21). A list of SPOUT methyltransferases, the modifications they incorporate, and other molecular features discussed throughout this review is shown in Table 1. The locations of selected example modifications are also shown on their respective RNA structures in Figure 1, highlighting the diversity of RNA methylations, including m^1^R (R = purine, A or G), m^1^Ψ (Ψ = pseudouridine), m^3^Ψ/U, and 2′-O-methylation (Nm; N = any nucleotide, A, U, C, G) (23, 24, 25, 26, 27).

**Table 1:** Properties, substrates, and modifications incorporated by SPOUT methyltransferases included in the phylogenetic analysis and discussed throughout this review

Clade^a^	Enzyme	PDB	Organism^b^	Substrate (Modification)^c^	Dimerization mode	Domain architecture	Domain length d		
							SPOUT	NTD	CTD
1	TrmD	4YVG, 4YVI^e^	B	tRNA (m^1^G_37_)	Antiparallel	SPOUT–CTD	160^f^	N/A	86
	TrmJ	4XBO	B/A	tRNA (Cm_32_/Um_32_)	Perpendicular	SPOUT–CTD	179^g^	N/A	67
	TrmL	4JAL	B/A	tRNA (Cm_34_; Um_34_)	Perpendicular	SPOUT only	157^g^	N/A	N/A
	TrmH	1V2X	B	tRNA (Gm_18_)	Perpendicular	NTD–SPOUT–CTD	155^h^	21	18
	Sfm1	5C77	E	r-protein eS3 (Ω-methylation Arg146)^f^	Monomer	SPOUT–CTD	154^i^	N/A	59
2	Trm56	2YY8	A	tRNA (Cm_56_)	Perpendicular	SPOUT–CTD	159^j^	N/A	44
	Nep1	3OII, 3OIJ^e^	A/E	16S m^1^Ψ_914_^g^; 18S rRNA m^1^Ψ_1189_^f^	Perpendicular	NTD–SPOUT	209^g^	42	N/A
	RsmE	4E8B	B/E	16S rRNA (m^3^U_1498_)^g^	Antiparallel	NTD–SPOUT	172^g^	71	N/A
3	Trm3	N/A	E	tRNA (Gm_18_)	Perpendicular	NTD–SPOUT	152^i^	1284	N/A
	MRM1	N/A	E	16S mitochondrial rRNA (Gm_1145_)^h^; 21S mitochondrial rRNA (Gm_2270_)^f^	ND^k^	NTD–SPOUT	171^l^	241	N/A
	RlmB	1GZ0	B	23S rRNA (Gm_2251_)^g^	Perpendicular	NTD–SPOUT	162^g^	81	N/A
	TsnR	3GYQ	B	23S rRNA (Am_1067_)^j^	Perpendicular	NTD–SPOUT	164^m^	105	N/A
	MRM3	7OI6	E	16S mitochondrial rRNA (Gm_1370_)^h^	ND^k^	NTD–SPOUT	211^l^	209	N/A
	AviRb	1X7P	B	23S rRNA (Um_2479_)^l^	Perpendicular	NTD–SPOUT	171^n^	116	N/A
	TrmY	N/A	A	tRNA (m^1^Ψ_54_)	Perpendicular	NTD–SPOUT or SPOUT–CTD	ND^k^	ND^k^	ND^k^
4	Trm10	4JWJ, 7ONU^e^	A/E	tRNA (m^1^G_9_ and/or m^1^A_9_)	Monomer	NTD–SPOUT–CTD	158^o^ 192^i^ 191^l^ 192^l^ 202^l^	88 84 94 116 142	46 17 54 8 20
	RlmH	5TWJ	B	23S rRNA (m^3^Ψ_1915_)^g^	Antiparallel	SPOUT only	155^g^	N/A	N/A

a Representative clade for each SPOUT methyltransferase based upon the phylogenetic tree in Figure 2.

b Enzyme found in organisms among Bacteria (B), Archaea (A), and/or Eukarya (E).

c Because the numbering for sites of modification are not conserved for rRNA from different organisms, the organism is indicated for each rRNA modification site.

d Representative examples of domain lengths (amino acids) from organisms that have been characterized structurally or by multiple sequence alignment. If the length of the linker region connecting the SPOUT domain to its extended domain has been identified in the SPOUT methyltransferase structure, the numbering is included as part of the respective extended domain. Domain architecture for TrmY is not clear from available sequences.

e Structure includes RNA substrate.

f *Haemophilus influenzae*.

g *Escherichia coli*.

h *Thermus thermophilus*.

i *Saccharomyces cerevisiae*.

j *Pyrococcus horikoshii*.

k ND: Not determined.

l *Homo sapiens*.

m *Streptomyces azureus*.

n *Streptomyces viridochromogenes*.

o *Sulfolobus acidocaldarius*.

**Figure 1:**
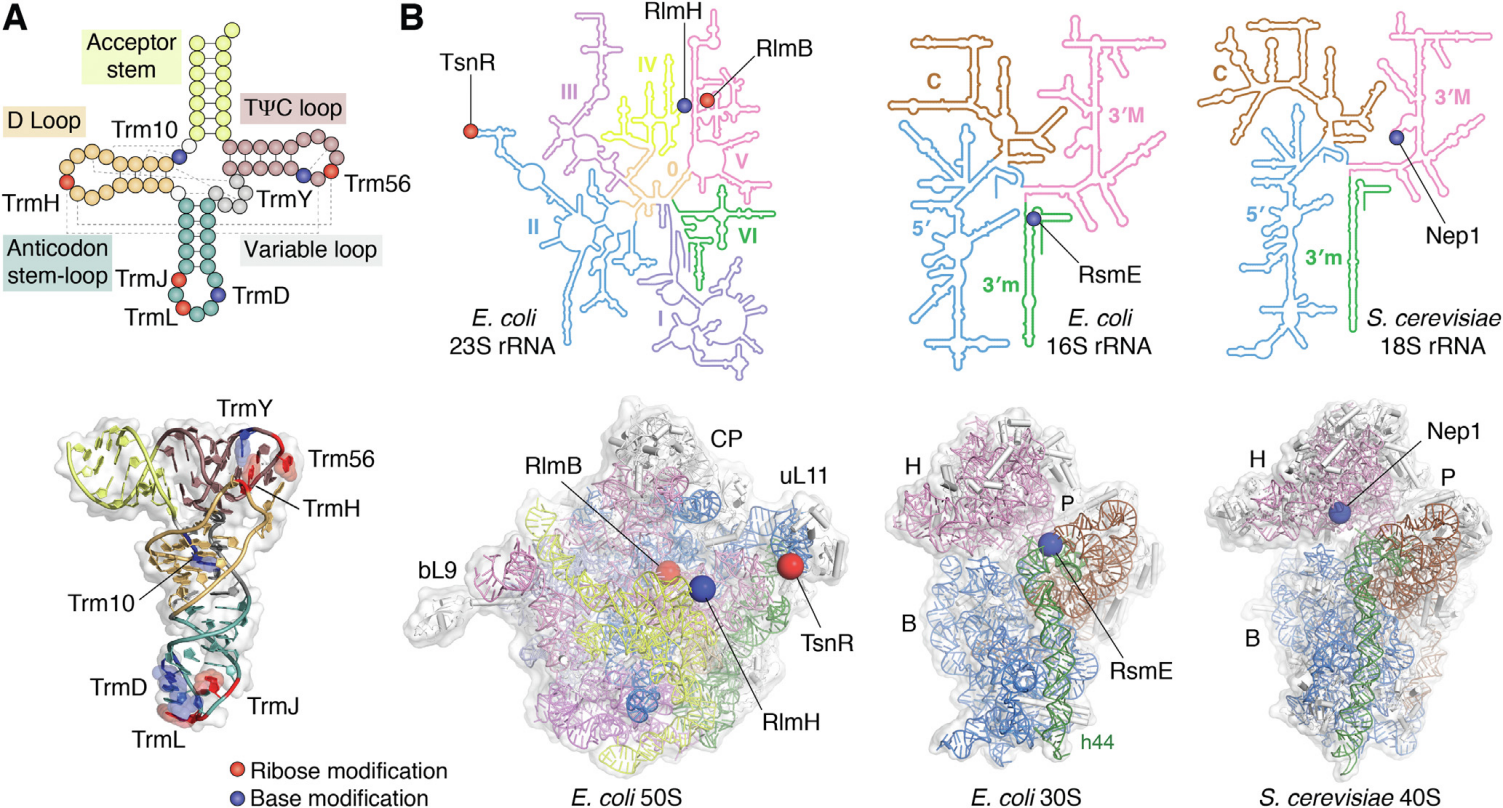
**RNA SPOUT methyltransferase target sites in tRNA and rRNA.***A*, sites of modification by tRNA-modifying SPOUT methyltransferases mapped onto the tRNA secondary (*top*) and tertiary (*bottom*) structures (shown on tRNA^Phe^, PDB 6LVR). Each modification is colored based on the type of modification (*red*: ribose modification; *blue*: base modification) and labeled with the SPOUT methyltransferase responsible for the modification. Tertiary interactions which form the L-shaped three-dimensional structure are shown as *dotted lines* on the secondary structure. *B*, sites of rRNA modification by SPOUT methyltransferases mapped onto the rRNA secondary structure (*top*) and structures of the applicable ribosomal subunit (*bottom*; shown for *E. coli* 30S and 50S (PDB 4V4Q) and *S. cerevisiae* 40S (PDB 4V88), as indicated). rRNA secondary structure maps were adapted from http://apollo.chemistry.gatech.edu/RibosomeGallery under a CC BY-SA 3.0 license (112). Ribosome structural features noted on the structures are central protuberance (CP), bacterial ribosomal protein L9 domain (bL9), universal ribosomal protein L11 domain (uL11), head (H), platform (P) and body (B). SPOUT, SpoU-TrmD.

Modifications by SPOUT methyltransferases are important for all three domains of life and play key roles in RNA function by impacting RNA stability, ribosomal fidelity, and bacterial antibiotic resistance. For example, tRNA methylation by SPOUT methyltransferases provides stability to tRNA through effects on structure (*e.g.*, Gm18, TrmH) and can be essential for fidelity of decoding, such as by preventing ribosomal frameshifting (*e.g.*, TrmD, m^1^G37) (4, 5, 28, 29, 30, 31, 32). More recent developments have revealed that tRNAs and their modifications play global roles in biological systems beyond simply ensuring tRNA stability or optimal structure for translation. During cellular stress, tRNA modifications can be altered to regulate translation and gene expression, and tRNA fragments are increasingly implicated in diverse processes, such as cell signaling and stress response (33, 34). New links have also been described between tRNA modifications and disease, particularly metabolic and neurological disorders and cancer (35, 36, 37). Many tRNA modifications are performed at the same position by nonhomologous enzymes in bacteria and eukaryotes, making them potentially interesting targets for antibacterial drugs. A better understanding of bacterial SPOUT methyltransferases could therefore prove important in the age of increasing antibiotic resistance. Other tRNA modifications and modification enzymes could prove useful in drug design, as evident from the immunostimulatory role of Gm18 modification (performed by TrmH) (38).

rRNA modifications placed by SPOUT methyltransferases hold essential functions in ribosome maturation, such as ribosome assembly and biogenesis, and modifications to the peptidyl transferase center and the decoding site aid in accurate translation (39). In some bacteria, lack of modifications to 16S rRNA disrupts formation of the 30S ribosomal subunit and binding of initiator tRNA (40, 41). SPOUT methyltransferase Nep1, for example, is required for assembly of the small ribosomal subunit, and mutation of the enzyme is linked to Bowen–Conradi Syndrome in humans (42, 43). The bacterial ribosome is also a major target for antibiotics, and rRNA methylation is a tool exploited by many bacteria to gain antibiotic resistance by sterically blocking antibiotic binding (44). Methylations incorporated by intrinsic or acquired methyltransferases, including some members of the SPOUT superfamily, can confer exceptionally high-level antibiotic resistance. For example, the thiostrepton-resistance and avilamycin-resistance SPOUT 2′-O-methyltransferases, TsnR and AviRb respectively, modify distinct functional regions of the 23S rRNA to sterically block antibiotic binding and eliminate antibacterial activity (45, 46).

With these important roles in biology—and likely many more that remain to be elucidated—characterization of SPOUT methyltransferases and their mechanisms of action is critical. Interestingly, despite extensive structural and biochemical characterization of several SPOUT family members, there are few common themes that have emerged to date, beyond the active site trefoil knot that serves as a conserved and defining feature. Although Class I methyltransferases are more diverse with their ability to modify a wide array of DNA, RNA, or protein substrates, the SPOUT methyltransferase family is much smaller and yet has an incredible amount of mechanistic diversity considering that almost all enzymes within the family act on an RNA substrate. SPOUT methyltransferases have relatively little conservation between different family members in terms of primary sequence, overall domain structure, catalytic mechanism, or mode of RNA binding and recognition. For example, some SPOUT methyltransferases only discriminate substrate at a post binding step, allowing methyl transfer to occur for substrate only (*e.g.*, Trm10), while others (*e.g.*, TrmH) only bind and methylate their specific substrates (28, 47). These distinct features illustrate the fascinating biochemical and mechanistic diversity of this enzyme superfamily. In this review, we provide a new maximum likelihood (ML)-based phylogenetic analysis of the SPOUT superfamily as a basis to compare similarities and differences in enzyme structure, domain organization, and key elements of catalytic function, including SAM binding and substrate recognition.

## Phylogenetic analysis of the SPOUT methyltransferase superfamily

The SPOUT methyltransferases adopt a characteristic α/β knotted fold but show a high level of sequence diversity, making accurate phylogenetic analyses a challenge. A previous study used 15 representative SPOUT enzymes from the Clusters of Orthologous Groups database as seeds to generate a homologous sequence set for phylogenetic tree construction (17). However, the common tree reconstruction techniques of neighbor-joining, maximum parsimony, and ML either did not generate a tree with well resolved branches and high support values or were computationally impractical at that time (for ML). We re-addressed this challenge using the much larger sequence dataset (276,000 sequences) now available in the InterPro database (ID: IPR029028). Specifically, our goal was to infer the phylogenetic relationship of the evolutionarily conserved SPOUT domain across the entire family of SPOUT methyltransferases, omitting the N-terminal domain (NTD) and C-terminal domain (CTD) extensions which are likely to have been acquired by these enzymes through independent evolutionary events.

**Figure 2:**
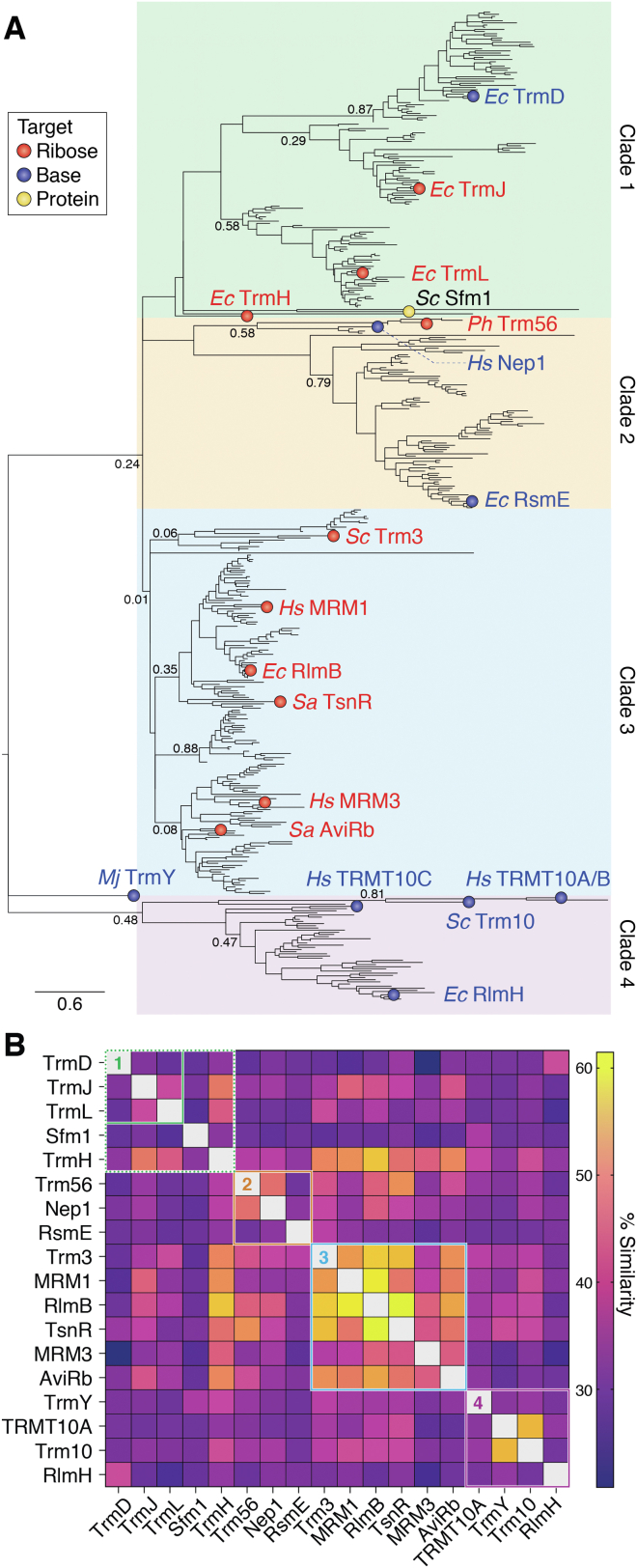
**Phylogenetic analysis of the SPOUT superfamily.***A*, the maximum likelihood phylogenetic tree of SPOUT domains from diverse methyltransferases. The tree is clustered into four major clades (Clade 1–4), and enzyme names are colored to indicate the type of modification. Bootstrap values are noted on select branches to highlight the low support for most deep nodes but high support for the terminal nodes and thus composition of individual subclades. *B*, heat map comparison of sequence similarity (% similarity) for the SPOUT domain of the indicated SPOUT methyltransferases. Major Clades (1, 2, 3, 4) from the tree are indicated by the color-coded boxes. The sequence similarity was calculated in the Geneious software based on BLOSUM 45 scoring matrix allowing consideration of similarities in residue physical or chemical properties. SPOUT, SpoU-TrmD.

Most of the currently available 276,000 SPOUT domain-containing sequences are found in bacteria (248,000), with Eukarya and Archaea having 15,000 and 9000 sequences, respectively. Using the UniRef50 dataset (*i.e.*, representative sequences with less than 50% sequence identity), these diverse protein sequences were aligned based on their SPOUT domain only and used to create a phylogenetic tree by the ML method (Fig. 2*A*). For the phylogenetic reconstruction, only the SPOUT domain sequences were considered, as these homologous sequences should contain essential residue signatures, while inclusion of NTD or CTD sequence would result in poor alignment and unrealistic phylogenetic inferences. ProtTest (48) was used to determine the best fit model of amino acid substitution based on Akaike information criterion, and the phylogenetic tree was bootstrapped 100 times. This new dataset includes a greater number of Clusters of Orthologous Groups compared to the previous analysis, including multiple superfamily members for which functional information is available (enzymes indicated on the tree in Fig. 2*A*). As observed previously (17), our ML tree has low bootstrap values in most deep nodes, while the terminal nodes have high values giving strong support to the composition of individual subclades. Further, we also used a BLOSUM45 similarity matrix of representative SPOUT methyltransferases (indicated in Fig. 2*A*) to corroborate clustering in the ML phylogenetic tree with members of the same clade typically showing higher similarity values compared to those outside (Fig. 2*B*). Two exceptions to this are TrmH and Sfm1 which have broader similarity or dissimilarity scores with members of multiple clades, respectively, and thus are not confidently assigned to one of the major groups. In our multiple sequence alignment dataset, the lowest amount of pairwise identity is ∼4%, while the overall average identity as calculated by the ALISTAT server is 16%.

Our new ML phylogenetic tree reveals four major groupings (Clades 1–4; Fig. 2*A*) of SPOUT-domain methyltransferases, with numerous subclades mostly containing at least one functionally characterized enzyme. Most of the enzymes within Clade 1 modify tRNAs in their anticodon stem-loop. These enzymes include the tRNA methyltransferases TrmJ and TrmL, which modify ribose 2′-OH, and TrmD, which methylates a guanosine base. From the phylogenetic tree, TrmD appears to have evolved later than the ribose 2′-OH methyltransferases of the same clade. We also observe a distinct branch that is most closely associated with Clade 1 and which includes another tRNA modifying SPOUT enzyme, TrmH. In contrast to the Clade 1 enzymes noted above, however, TrmH methylates tRNA outside the anticodon stem-loop. This distinct branch also includes the protein methyltransferase Sfm1, indicating it may have a most recent common ancestor with SPOUT superfamily enzymes of Clade 1. Characterized SPOUT methyltransferases in Clade 2 include Nep1, Trm56, and RsmE, with the RsmE methyltransferases more distant phylogenetically from Nep1 and Trm56. Nep1 and RmsE are both rRNA methyltransferases and modify a pseudouridine base in 18S rRNA and uridine base in 16S rRNA, respectively. However, these enzymes are mechanistically distinct in their action, acting on free rRNA (Nep1) and assembled 30S subunit rRNA (RsmE), perhaps reflecting their evolutionary distance within Clade 2. In contrast to both other examples in Clade 2, Trm56 methylates the ribose 2′-OH on a tRNA substrate. Clade 3 represents a large group of diverse methyltransferases, including Trm3, MRM1, MRM3, TsnR, AviRb, and RlmB, all of which methylate the ribose 2′-OH at different locations within tRNA or rRNA. Given this common modification type for all clade members, these enzymes likely evolved from an ancestral SPOUT 2′-OH methyltransferase. Finally, Clade 4 consists of methyltransferases which diverged from a common ancestor much earlier and includes all Trm10 enzymes and RlmH, which all methylate RNA on the nucleobase.

In summary, this phylogenetic analysis gives a glimpse into the possible phylogenetic relationships between diverse SPOUT superfamily members that have been difficult to rationalize due to the diverse functional features associated with these enzymes, as described in more detail in this review. However, detailed insight into the overall evolution of the SPOUT superfamily remains limited by high sequence divergence, low support in the deep tree branches, and potential influence of the NTD/CTD sequences which were excluded from the alignment due to their even greater sequence and structural divergence. For example, as all sequences in the phylogenetic tree possess less than 50% sequence identity, predictions for enzymes of unknown function within each clade should be made with caution. While details such as the specific site of base modification would be highly speculative based on the phylogeny alone, new inferences on likely substrate or target (*e.g.*, rRNA *versus* tRNA or 2′-OH *versus* base modification) might reasonably be made based on phylogenetic closeness to a known representative.

## SPOUT methyltransferase structure, SAM binding, and domain organization

**Figure 3:**
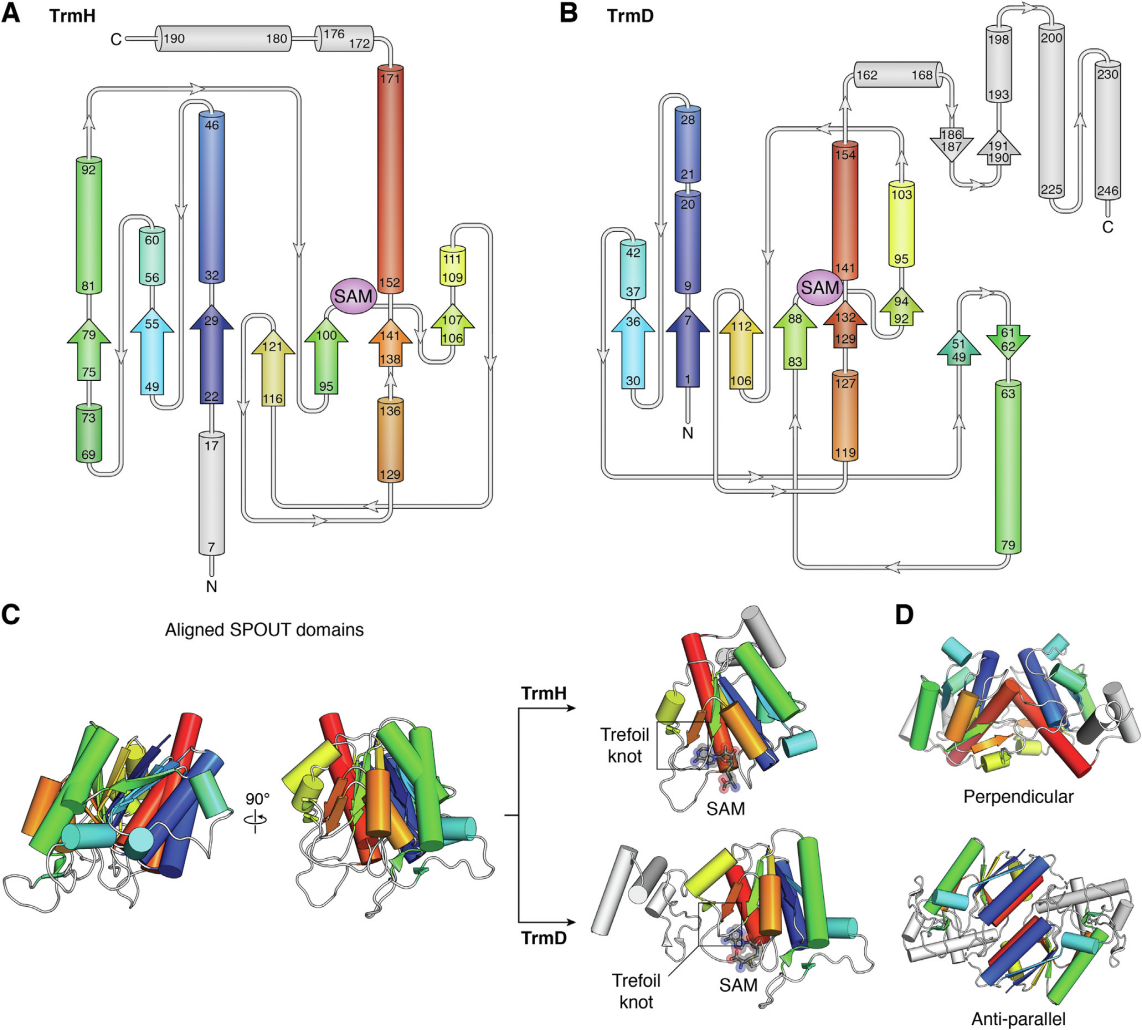
**Overview of SPOUT methyltransferase structure.** Topology maps of *A*, TrmH (derived from *T. thermophilus*; PDB 1V2X) and *B*, TrmD (derived from *H. influenzae*; PDB: 4YVG) are shown with α-helices represented by *cylinders* and β-sheets indicated by *arrows*. In both maps, the SAM-binding region is shown. The SPOUT domain is colored as a rainbow gradient (*blue to red*) from N- to C-terminus, with NTD and CTD extensions shown in *gray*. *C*, two orthogonal views of the structures of TrmH and TrmD aligned by their SPOUT domain β-sheets (*left*) and the individual protomers of TrmH (*top right*) and TrmD (*bottom right*). The trefoil knot and the position of the bound cofactor SAM are indicated. *D*, perpendicular and antiparallel dimerization modes exemplified by TrmH (*top*) and TrmD (*bottom*); note the distinct, characteristic orientations of the *red* and *blue* α-helices in each dimer. CTD, C-terminal domain; NTD, N-terminal domain; SAM, S-adenosyl-L-methionine; SPOUT, SpoU-TrmD.

The SPOUT domain consists of a protein backbone (∼160 amino acids) which is passed three times in and out of a loop to form a topological trefoil knot in its C-terminal region (16, 18, 21) (shown using TrmD and TrmH as examples in Fig. 3). This characteristic feature of SPOUT methyltransferases has less sequence variation (is more conserved) as compared to other regions of the SPOUT domain, especially within each clade of our phylogenetic tree. The average sequence identity for the full alignment is 16%, while the region corresponding to the trefoil knot exhibits 28% identity. Further, there is 94 to 99% conservation of glycine residues within the knot region among highly diverse methyltransferases, showing the role of specific sequence as well as structural conservation in this defining feature of the SPOUT domain.

**Figure 4:**
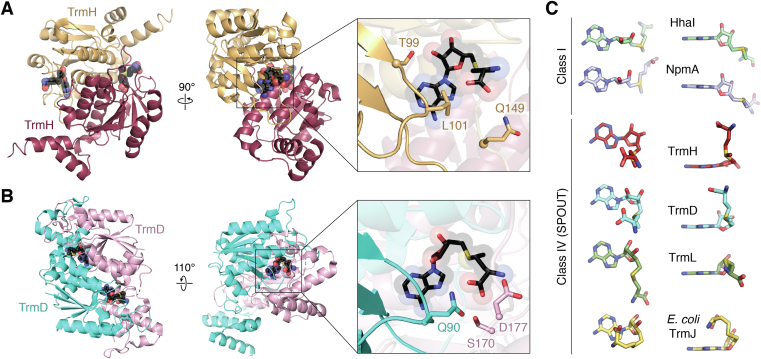
**SAM conformations and SAM-binding pockets of representative SPOUT methyltransferases.***A* and *B*, structural overview of the TrmD and TrmH dimers, respectively, showing a zoomed-in view of the SAM-binding pocket of each enzyme (*boxed*, *right*). The zoomed-in view highlights the proximity of the bound SAM to the characteristic SPOUT knot (*solid cartoon*), dimer interface (*transparent cartoons*, colored as in the structural overviews), and key residues whose side chains interact directly with SAM (shown in *black* in both structures). *C*, the elongated conformation of SAM in two representative class I methyltransferases, HhaI (PDB 2HMY) and NpmA (PDB 3MTE), is shown for comparison to the characteristic bent conformation in class IV SPOUT methyltransferases, exemplified by TrmD (PDB 4YVG) and TrmH (PDB 1V2X). Two additional, distinct SPOUT cosubstrate binding modes are also shown: a more extended form observed for one protomer in the TrmL dimer (PDB 4JAL) and the superbent conformation observed in *E. coli* TrmJ (PDB 4CNE). For both the TrmL and TrmJ structures, SAH was bound in the cosubstrate binding pocket. SAM, S-adenosyl-L-methionine; SAH, S-adenosyl-homocysteine; SPOUT, SpoU-TrmD.

Knots are known to provide stability to protein structure and resistance to degradation (49, 50) and, in the case of SPOUT methyltransferases, the trefoil knot also provides the binding site for the essential SAM cosubstrate (51) (Fig. 4, *A* and *B*). Cosubstrate binding at this unique structural feature promotes methyl transfer by orienting groups within the active site in an optimal conformation (49, 50). The bound SAM adopts a unique bent conformation in SPOUT methyltransferases with its methionine moiety rotated 80° to face the adenosine component (Fig. 4*C*). In contrast, when bound to other SAM-dependent enzymes these groups are extended ∼180° away from one another (Class I) or the methionine group is rotated ∼90° in the opposite direction (Classes II, III, and V) (1, 12, 50). Analysis of TrmD structures along with molecular modeling revealed that when the trefoil knot is missing, SAM cannot adopt the bent conformation in the enzyme active site (50). SAM is consequently positioned in a nonoptimal extended conformation in which the methyl group is further from the target atom, and there is a steric clash between SAM and the tRNA substrate. The presence of the trefoil knot thus enforces the bent SAM conformation, prevents steric clashes, and optimally positions the methyl group relative to the substrate for transfer.

Almost all SPOUT methyltransferases function as homodimers with the active site forming upon dimerization to bind two SAM molecules, but only one RNA substrate. Along with the SAM-bound trefoil knot of the SPOUT domain, a four-helix bundle forms at the dimer interface with two α-helices from each protomer assembling in a perpendicular (rotated ∼90° from one another) or an antiparallel fashion (rotated ∼180°; Fig. 3*D*). Each dimerization mode tends to align with a specific type of RNA methylation, with the perpendicular and antiparallel dimerization modes corresponding most often to ribose sugar or base modification, respectively (Table 1) (17). Finally, Trm10 and Sfm1 are distinct from other SPOUT methyltransferases as they function as monomers, reminiscent of Class I (Rossmann-like fold) methyltransferases, despite having the SPOUT-defining trefoil knot in their active sites (20, 52, 53). Our phylogenetic analysis indicates that these enzymes may have evolved from ancestral dimeric proteins by loss of the dimeric interface (Fig. 2).

SPOUT methyltransferases can be composed of the SPOUT domain alone or have an extended NTD and/or CTD surrounding the SPOUT domain (10, 21). Extended sequences vary drastically among SPOUT methyltransferases, ranging from very short (<20 amino acids) to over 1000 amino acids in length. Examples of SPOUT methyltransferases with each possible configuration of domain structure have been identified and characterized: SPOUT domain only (*e.g.*, TrmL and RlmH), N-terminal extension only (*e.g*., RsmE and TsnR), C-terminal extension only (*e.g.*, TrmJ, TrmD, and Smf1), and both N- and C-terminal extended (*e.g.*, Trm10 and TrmH) (Table 1). Interestingly, each domain structure subgroup contains at least one enzyme that modifies RNA at the ribose 2′-OH and one that modifies at the base. Though the SPOUT domain binds cosubstrate SAM and, in some instances, can discriminate between different modification targets (28), SPOUT methyltransferases with extended domains have been proposed to use these extra sequences to aid in protein dimerization, RNA binding, and/or methylation of their substrate pool (21). As the SPOUT methyltransferase superfamily evolved, diversification of the SPOUT methyltransferases, presumably driven by expansion of target substrates, resulted in loss or gain of these N- and/or C-terminal appendages.

The minimalist SPOUT methyltransferases, which lack any appended domains, must contain all residues and structural features necessary for specific substrate recognition and methylation within the SPOUT domain itself. The most well-characterized minimalist SPOUT enzymes are TrmL (YibK) and RlmH (YbeA), which methylate the ribose 2′-OH and nucleobase, respectively (54, 55). Although both contain the SPOUT domain and use dimerization to form their active sites, they act at unique positions on their respective tRNA and rRNA substrates providing the clearest example that SPOUT domain sequence variation and dimerization mode alone are sufficient to drive unique methylation abilities (23, 56).

Two well-characterized SPOUT methyltransferases with only NTD extensions are TsnR (2′-OH methylation) and RsmE (base methylation). The NTD extensions of TsnR and RsmE are structurally similar to ribosomal protein eL30 and PUA (pseudouridine synthase and archaeosine-specific transglycosylase) RNA-binding domains, respectively, highlighting their likely importance in RNA binding (46, 57). In both instances, the extended domain is also essential for substrate methylation to occur efficiently, as discussed further in the following sections. SPOUT methyltransferases with CTD extensions again include both a ribose 2′-OH (TrmJ) and a base (TrmD) modifying RNA methyltransferase, as well as Sfm1 which acts on a protein substrate (20, 27, 29). TrmJ is inactive without its CTD extension, while mutation of critical residues in the TrmD or Sfm1 CTD abolishes methylation by these enzymes (20, 24). Despite sharing a common domain organization, these three enzymes show that the presence of a domain extension alone does not enforce a particular quaternary structure or the identity of the substrate to be methylated. Finally, SPOUT methyltransferases with both an extended NTD and CTD around the SPOUT domain include Trm10 and TrmH (19, 25). The 2′-OH methyltransferase TrmH is evolutionarily distinct from base methyltransferase Trm10 despite their shared domain architecture (Fig. 2). TrmH and Trm10 also have far more diversity in their extended domain structures among the homologs of each enzyme than other SPOUT methyltransferases (21, 28, 52).

A central question to consider when characterizing SPOUT methyltransferases is how this superfamily of enzymes with a common SPOUT domain structure acts on diverse substrates in unique mechanistic ways, while also taking advantage of all the differences that have been identified in external domains, dimerization mode, and/or sequence. Furthermore, some close relatives of the same SPOUT methyltransferase have similar domain structure and yet have distinct mechanisms, attesting to a combination of currently ill-defined factors that define overall methyltransferase activity. Nonetheless, recent advances in structural and biochemical characterization of SPOUT enzymes have revealed many functional and mechanistic intricacies for each type of SPOUT methyltransferase, and these features are described in more detail below.

## Substrate recognition and modification by SPOUT methyltransferases

Correct substrate recognition is an essential step for enzyme specificity that involves accurate discrimination between the correct target molecule at its modification site and other structurally similar molecules. SPOUT methyltransferases have strict substrate specificity with each enzyme acting only on a specific subset of RNAs or protein and at a single or very limited number of modification sites. Despite their shared catalytic SPOUT domain, there is considerable variation in the mechanism of substrate recognition between members of the SPOUT superfamily and, complicating a deep understanding of these processes, even between direct homologs of the same enzyme from different organisms.

As noted already, RNA-modifying SPOUT methyltransferases act on either the ribose 2′-OH or various sites on the nucleobase. Through apparent parallel evolution of various structural and domain organization features within each subgroup of SPOUT methyltransferase, significant variation in the mechanism of substrate recognition has arisen, including differences in target nucleotide specificity and recognition of distinct RNA structural elements. In the following sections, we discuss features of substrate recognition by SPOUT methyltransferases and highlight our current understanding of both similarities and differences within this diverse pool of enzymes. Enzymes are organized by modification type (ribose 2′-O-methylation, base methylation, and protein methylation) with each subsection ordered by clade (Clade 1–4).

### Ribose 2′-O-methylating SPOUT RNA methyltransferases

Multiple SPOUT methyltransferases catalyze ribose 2′-O-methylation of target nucleotides in either tRNA or rRNA. Although these enzymes modify the ribose common to the four nucleotide bases, the base identity of the target nucleotide can differentially impact methylation activity of individual SPOUT enzymes, highlighted by experiments where target nucleotides were mutated without affecting overall substrate structure. From our phylogenetic analysis, this nucleotide specificity does not seem to be a monophyletic trait, indicating that this family of enzymes did not evolve uniformly over time to become more or less specific with respect to target nucleotide recognition. Ribose 2′-O-methylating SPOUT methyltransferases are spread out across three different clades with Clade 1 containing two enzymes (TrmJ and TrmL) and being most closely associated with a third (TrmH), Clade 2 containing one (Trm56), and Clade 3 containing the majority (Trm3, MRM1, RlmB, TsnR, MRM3, and AviRB) (Fig. 2). As discussed below for the best characterized enzymes, ribose 2′-O-methylating enzymes have been observed to recognize distinct features in their substrate, including, to varying extents, contacts with more distant structural elements.

#### TrmJ

TrmJ methylates tRNA at position 32, within the anticodon stem-loop (24, 27). This SPOUT methyltransferase has a CTD extension connected to the SPOUT domain *via* a 16 amino acid linker sequence (23). The domain lengths of TrmJ homologs across organisms are fairly consistent, with the SPOUT domain and CTD extension consisting of ∼180 and ∼70 amino acids, respectively (Table 1) (58). The CTD contains positively charged residues which aid in RNA binding, although the CTD alone cannot efficiently bind substrate tRNA (58). Deletions of different regions of the protein—CTD, SPOUT domain, or part of the linker—uncovered that each is essential for methylation activity (24, 58). Additionally, swapping the CTDs of TrmJ enzymes from different species resulted in loss of methylation activity, despite their similar sizes. Therefore, although these methyltransferases perform the same modification in their respective organisms, each has its own specific CTD dependency to maintain methyltransferase activity. These differences between family members clearly play vital roles in diversifying the substrate pool despite similar domain architecture.

*Escherichia coli* TrmJ is the only SPOUT methyltransferase identified to date that can modify the ribose of tRNA position 32 regardless of the identity of the nucleotide. Although Cm32 and Um32 appear to be the only physiologically relevant modifications introduced (24), this implies that *E. coli* TrmJ does not recognize the nucleotide base at the site of modification. This differs from *Sulfolobus acidocaldarius* TrmJ which is only able to modify a cytidine at the same position. One clear distinction identified between these homologs that might explain these distinct specificities appears to be the differing conformations of the bound co-substrate in each TrmJ enzyme (discussed in more detail later). Another distinction between the two TrmJ orthologs involves overall recognition of the tRNA substrate: *S. acidocaldarius* TrmJ can effectively modify a truncated tRNA structure corresponding to the anticodon stem-loop fused to an acceptor stem (24, 58), whereas *E*. coli TrmJ also requires the D- and T-arms within the full tertiary tRNA structure. The different requirements for tRNA substrate recognition may be due to different sizes of the positively charged area in the cleft of the dimer interface. However, the current lack of a structure of the tRNA-bound enzyme precludes detailed understanding of these or other differences that may contribute to the distinct TrmJ nucleotide specificities.

#### TrmL

TrmL is a minimalist SPOUT methyltransferase that modifies the first anticodon nucleotide (position 34) of tRNA (54). TrmL functions as a homodimer with dimerization being essential to form a stable complex with substrate tRNA: a Tyr142 to alanine substitution disrupts dimer formation and eliminates the ability of TrmL to bind tRNA (23).

TrmL exhibits some flexibility in target nucleotide selection with the ability to methylate modified 5-carboxymethylaminomethyluridine (cmnm^5^U), unmodified U, or unmodified C at position 34 (54). Additionally, A35 is a key residue for substrate recognition by TrmL, and A36-A37-A38 are important either *via* direct interaction with TrmL or due to the necessity for prior isopentenylation (i6) on A37 (59). As such, TrmL is one of the few SPOUT enzymes that requires a prior modification to the substrate before methylation can occur. The i6 modification on A37 has been hypothesized to guide TrmL methylation by increasing the chance of nucleotide 34 having direct interaction with the enzyme (23, 59). TrmL is also one of relatively few SPOUT methyltransferases that can efficiently modify a truncated tRNA structure (59), requiring only an anticodon stem-loop minihelix with an extension of two base pairs. A high-resolution structure of TrmL bound to tRNA will be an important future step to help elucidate the determinants of specific substrate recognition.

#### TrmH/Trm3

TrmH and Trm3 catalyze the Gm18 modification in the D-loop of tRNA, in Bacteria and Eukarya, respectively (19, 60, 61). Among TrmH enzymes, there is considerable variation in the size and configuration of appended domains. *Thermus thermophilus* and *Aquifex aeolicus* TrmH have similar sized NTDs and CTDs (∼20 amino acids) with extended α-helices surrounding the SPOUT domain (28), while the CTD of *E. coli* TrmH is >30 amino acids longer and forms a structure comprising one α-helix and three β-strands (28). In even starker contrast, eukaryotic Gm18 modifying enzymes such as *Saccharomyces cerevisiae* Trm3 and human TARBP1 have extremely long NTDs (1280 and 1400 amino acids, respectively) but no CTD extensions (62, 63). Although these eukaryotic homologs are fully active without CTD extensions, deletion of the *T. thermophilus* TrmH CTD renders the enzyme unable to bind and methylate tRNA (28). Additionally, the NTD of TrmH in *T. thermophilus* plays an important role in stabilizing the homodimer structure and was found to be important for protein stability, thus making the NTD necessary for both methylation activity and tRNA-binding (16, 28, 64). The apparent distinct evolutionary origin of TrmH and Trm3 enzymes (Fig. 2) may have contributed to the diverse array of domain structures and functions observed within enzymes that catalyze the Gm18 modification and underscores the complexity present even within SPOUT enzymes that catalyze identical modifications.

Diverse TrmH homologs also exhibit distinct RNA substrate specificities. For example, *T. thermophilus* TrmH can methylate all tRNA species while other TrmH homologs, for example from *A. aeolicus* and *E. coli*, can only modify a subset of tRNA with a G nucleotide at the target position 18 (65). Superposition of the SAM-binding domains of TrmH from *T. thermophilus* and *A. aeolicus* reveals a difference in the orientation of the α1/α8 extensions, and *A. aeolicus* TrmH contains a stretch of basic residues on this extension that is not found in the *T. thermophilus* enzyme (66). This region may therefore be responsible for the restricted specificity exhibited by *A. aeolicus* TrmH. Further, TrmH chimeras created by swapping CTD, SPOUT, and NTD domains between *T. Thermophilus* and *E. coli* family members produced enzymes with altered substrate specificities (28). These studies revealed that although the CTD and NTD play important roles in RNA binding, the SPOUT domain is primarily responsible for substrate recognition among studied enzymes in the TrmH family.

In the process of specific tRNA recognition by TrmH, the G18-G19 dinucleotide at the target site appears to be the only essential sequence determinant among the 18 conserved or semiconserved nucleotides identified among tRNA substrates; mutation of either guanosine nucleotide results in loss of methylation (67). The strict recognition of guanosine by TrmH also means that when G18 is mutated, TrmH can methylate the adjacent G19 instead. More specifically, the O^6^ atom of the guanine nucleobase is a positive determinant for target site recognition, considering that TrmH can methylate a tRNA with an O^6^-containing inosine at position 18 (65). TrmH from both *T. thermophilus* and *A. aeolicus* can modify a tRNA 5′ fragment with only the intact D-loop structure, although the reaction is considerably less efficient than for full-length tRNA (68, 69). Mutations that disrupt the tertiary base pairs between the D-loop and T-loop decrease binding of TrmH to tRNA significantly (67). This suggests that while the D-loop contains critical positive determinants for substrate recognition, ultimately the full tRNA tertiary structure including intact D-loop and T-loop interactions is required for optimal methylation activity. These findings also suggest that apart from the essential dinucleotide sequence at the target site, TrmH recognizes RNA backbone geometry as opposed to specific nucleotide sequences in the full-length tRNA structure (67).

#### Trm56

Trm56 modifies cytosine at nucleotide 56 in the T-loop of tRNA (70, 71, 72). Methylation activity is abolished when C56 is mutated to G, indicating that the identity of the nucleotide at the modification site is essential for recognition by Trm56 (71). Characterized Trm56 enzymes typically have CTD extensions of similar lengths appended to the SPOUT domain (Table I), though the *Thermoplasma acidophilum* Trm56 CTD is much greater in length with a HD (His-Asp) phosphodiesterase-like domain of almost 200 amino acids (73). Studies of Trm56 from *Pyrococcus abyssi* revealed this enzyme to be another example of a SPOUT methyltransferase which can act on a truncated tRNA, albeit with suboptimal methylation activity (70). Specifically, Trm56 can methylate the ribose of C56 in a stem-loop RNA corresponding to the isolated T-arm, but methylation is four- to five-fold less efficient than with the full-length substrate.

#### TsnR

The thiostrepton-resistance methyltransferase TsnR is a ribose 2′-OH modifying enzyme that methylates nucleotide A1067 located in a loop at the end of Helix 43 of the bacterial 23S rRNA (74). TsnR has an N-terminal extended domain that resembles the yeast RNA binding ribosomal protein eL30 and, like most SPOUT methyltransferases, TsnR functions as a homodimer (Table 1). The full-length enzyme has specific RNA binding that is of higher affinity than the SPOUT domain alone, while the NTD alone has no apparent binding affinity for RNA (46). The two domains thus appear to function in concert; the SPOUT domain initiates RNA binding which positions the NTDs for high affinity binding, substrate discrimination, and formation of a catalytically active complex. Notably, the substrate rRNA must undergo a conformational change led by the NTD that is required for catalysis; the isolated SPOUT domain cannot induce this conformational change and therefore cannot methylate substrate RNA despite having some intrinsic RNA affinity and containing the bound SAM co-substrate. In this instance, the extended domain is essential not only for binding but also catalytic activity.

TsnR specifically recognizes a U1066-A1067-G1068-A1070 loop sequence at the target site which caps 23S rRNA Helix 43 (75). Studies to elucidate the minimal substrate necessary for substrate recognition by TsnR determined that an isolated 29-nucleotide rRNA hairpin containing the target nucleotide acts as a more efficient substrate than the 58-nucleotide domain or full-length 23S rRNA (76). This enhanced substrate preference for the 29-nucleotide hairpin is most likely due to increased accessibility of the target nucleotide in the hairpin which lacks the complex tertiary structure of the full 58-nt rRNA domain. The nosiheptide-resistance methyltransferase, a close relative of TsnR, methylates the same site on the 23S rRNA and also displays similar specificities for a 29-nucleotide fragment (77). Interestingly, both TsnR and nosiheptide-resistance methyltransferase make a critical contact with nucleotide U1061, located in an internal loop within Helix 43 more distant from the target site, that allows for efficient substrate binding (76). This nucleotide makes interactions that stabilize the RNA tertiary structure of the 58-nt rRNA domain and, as a result, the protein–RNA contact may be required for RNA unfolding to fully expose the target nucleotide for recognition and modification.

### Base-modifying SPOUT RNA methyltransferases

SPOUT methyltransferases have been identified that modify both purine and pyrimidine nucleotide bases, generating m^1^Ψ, m^3^U/Ψ, or m^1^G/A modifications. Base-modifying SPOUT methyltransferases are found in Clade 1 (TrmD), Clade 2 (Nep1 and RsmE), and Clade 4 (Trm10 and RlmH); additionally, TrmY appears to have evolved independently from these other enzymes (Fig. 2). While most of these enzymes require dimerization for methylation activity, this subcategory contains Trm10 which is the only SPOUT RNA methyltransferase believed to be catalytically active as a monomer. The mechanisms of substrate recognition for base-modifying enzymes are known in some cases, but questions remain about the molecular details of substrate selection for others. The structures of TRMT10C, TrmD, and Nep1 in complex with their target RNAs have provided insight into some of the molecular contacts and structural features of the substrate that are exploited for specific recognition. However, these insights have also highlighted the need for additional structure-function studies to fully define how different members of this diverse enzyme family select and specifically modify their RNA substrate(s).

#### TrmD

Of all SPOUT methyltransferases, the mechanism of substrate recognition by TrmD has been investigated the most extensively. TrmD produces the m^1^G37 modification in the anticodon loop of tRNA in bacteria (29, 78). TrmD has a CTD extension following the SPOUT domain with a flexible linker connecting the two (Table 1) (18). The CTD is similarly sized (∼74–95 amino acids) across species where it has been characterized, to date (13). In the TrmD dimer, the SPOUT domain of one protomer and the CTD extension of the second jointly bind one tRNA at its anticodon branch. Additionally, the flexible interdomain linker becomes ordered and forms an α-helix when bound to tRNA (15). Both protomers in the TrmD dimer bind SAM, resulting in an enzyme–substrate complex comprising two SAM molecules but only a single tRNA per dimer, leaving the second active site nonfunctional. Residue Asp169 from the CTD extension is important for methyl transfer, as its mutation abolished TrmD methylation activity (15, 18, 51). Therefore, the SPOUT domain of TrmD alone is likely to be insufficient for binding and methyl transfer.

TrmD is highly dependent on nucleotide sequence at its target site, requiring the sequence G36-G37 for optimal tRNA methylation (79). Intriguingly, the dinucleotide GpG alone was found to be a minimal, albeit inefficient, substrate for TrmD. However, in *E. coli*, only a subset of tRNA with the G36-G37 sequence possess the m^1^G37 modification (80), indicating that GpG is a positive but not sufficient determinant for substrate recognition. Additional structural elements in tRNA are recognized by TrmD that can either make methylation more efficient or hinder the process. Subsequent studies revealed that TrmD from both *E. coli* and *A. aeolicus* can also methylate tRNA with the sequence A36G37, suggesting a more relaxed requirement of a purine nucleotide at position 36 (81). Notably, however, the A36-G37 sequence does not occur naturally in bacteria (15). In an engineered G36A tRNA variant, the 6-NH_2_ group of adenine most likely interacts with the carboxyl oxygen atoms of TrmD residue Asp50. However, the efficiency of methylation of transcripts with A36-G37 is lower, reflecting an overall K_M_ value that is slightly higher than that of wildtype tRNA (81, 82).

Although G36 and G37 are the only essential nucleotides for substrate recognition, TrmD recognizes additional structural elements throughout the tRNA anticodon loop. TrmD from *A. aeolicus* and *E. coli* can both modify an isolated 17-nucleotide anticodon stem-loop structure, although *E. coli* TrmD requires the addition of at least four base pairs for detectable methylation activity on the truncated substrate (79, 81, 83). Further, studies on *E. coli* TrmD reveal that although this homolog can modify a truncated tRNA transcript, the full-length tRNA is required for optimal catalytic efficiency (83).

**Figure 5:**
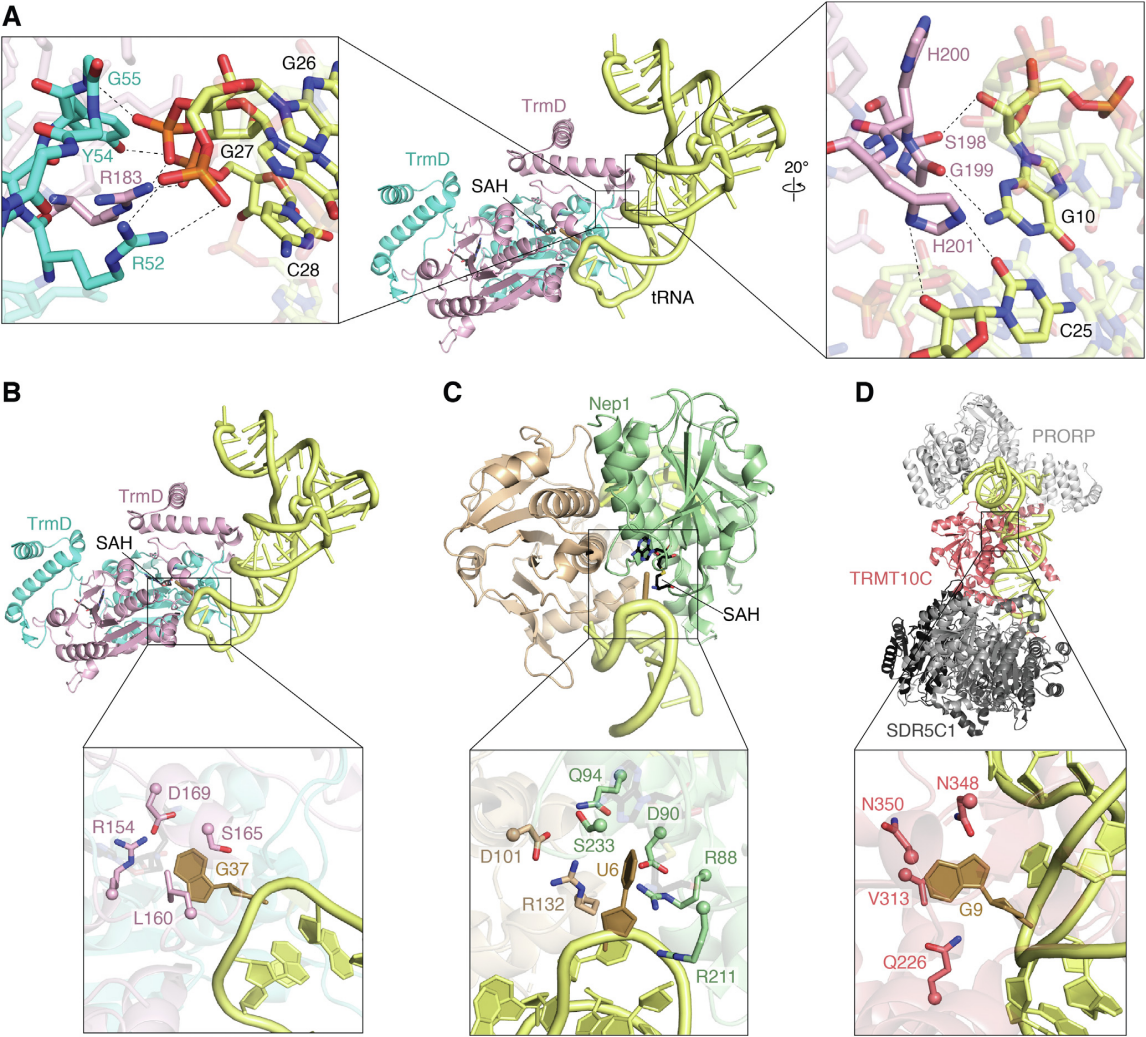
**Substrate recognition and base-flipping in SPOUT methyltransferase–RNA substrate complexes.***A*, key interactions involved in tRNA substrate recognition by TrmD including phosphate groups in the anticodon branch of tRNA (*left*) and critical contacts along the minor groove next to the G10:C25 pair (*right*) (PDB 4YVI). Target nucleotide base flipping observed in the structures of *B*, TrmD bound to substrate tRNA^Gln^ (PDB 4YVI), *C*, Nep1 bound to a model rRNA fragment (PDB 3OIJ), and *D*, TRMT10C as part of the mitochondrial RNase P complex with mitochondrial pre-tRNA^Tyr^ (PDB 7ONU; other protein components are shown in *gray*). In each structure, the target nucleotide (*gold*) is flipped into the binding pocket and stabilized by multiple protein residues (shown as *sticks*). For both TrmD and Nep1, the flipped base is sequestered at the SPOUT dimer interface. In all images, the RNA is shown in *yellow*. SAH, S-adenosyl-homocysteine; SPOUT, SpoU-TrmD.

While deletions of different tRNA regions resulted in reduced methylation activity, significant changes in tRNA sequence outside of the anticodon stem-loop had only modest effects on enzyme activity (81, 83). As noted for TrmH, this suggests that the primary RNA contacts may be with the phospho-sugar backbone of tRNA and that backbone geometry plays an important role in tRNA substrate recognition. Consistent with the previous biochemical data, the structure of the TrmD–tRNA substrate complex revealed essential contacts with the tRNA anticodon branch, comprising the D and anticodon arms and the variable loop (Fig. 5*A*) (15). However, TrmD does not directly contact the tRNA acceptor branch (acceptor and T arms), at apparent odds with the results of the previous methylation activity assays. These findings can be reconciled based on the essential nature of the overall tRNA structure for TrmD recognition. Specifically, while not directly contacted by the enzyme, the acceptor and T arms must be present for correct tRNA folding and thus for presentation of key determinants in the anticodon branch that are necessary for efficient methylation activity.

Uniquely, the methyl transfer reaction by TrmD only occurs when a Mg^2+^ is bound in the active site (84, 85). A recent study demonstrated through combined molecular dynamics simulations, quantum mechanical studies, and mutagenesis/enzyme activity assays that the essential Mg^2+^ ion binds to a negatively charged pocket in the TrmD active site, causing structural changes that force SAM to adopt its bent conformation and align with active site residues in an optimal orientation for catalysis (84). A previous metal rescue experiment suggested that the essential Mg^2+^ might interact with O^6^ of G37 (85), but the detailed computational studies suggest an alternative mechanism whereby the active site residue Arg154 stabilizes the O^6^ during the course of the methyl transfer reaction (84).

#### Nep1

Nep1 is a base modifying methyltransferase found in Archaea and Eukarya. Based on the RNA recognition sequence in yeast, Nep1 is predicted to act on Ψ1189 of 18S rRNA (43, 86, 87). Nep1 is highly dependent on recognition of the specific consensus sequence C/UUCAAC at the rRNA target site. This recognition is accomplished through base-specific interactions with protein residue side chains and peptide backbone in its binding pocket which have been characterized in detail through high-resolution crystallographic structural studies (88). As discussed later, Nep1 undergoes a structural rearrangement to accommodate the rRNA substrate, while also causing a conformational change in the RNA to flip out the target base for methylation.

#### RsmE

RsmE methylates U1498 to form m^3^U in a conserved region of helix 44 of bacterial 16S rRNA (89). RsmE includes an NTD extension, preceding its SPOUT domain, that is of similar length in most homologs (∼69–81 amino acids; Table 1). The NTD of *E. coli* RsmE is composed of five β-sheets and an α-helix and resembles the RNA-binding protein PUA (57). RsmE functions as a dimer with the PUA-like NTD of one protomer acting in RNA recognition and binding, and the SPOUT domain of the other presenting a bound SAM for methyl transfer. With its essential role in substrate binding, the NTD extension is thus required for methylation to occur (57). Studies to elucidate the minimal RsmE substrate uncovered that neither 16S rRNA nor 30S depleted of proteins serves as an efficient substrate. In contrast, mature 30S subunit is efficiently modified suggesting that a highly structured ribonucleoprotein particle late in the subunit assembly pathway is required (90). This substrate preference appears to be a general preference for methyltransferases that modify 16S rRNA near the ribosomal decoding center (91).

#### TrmY

TrmY modifies Ψ54 in the T-loop of tRNA and is another example of a SPOUT methyltransferase which is only able to act on a previously modified tRNA substrate, in which U54/U55 have been converted to pseudouridine by Pus10 (92). The necessity for modification prior to TrmY methylation hindered early efforts to characterize substrate recognition using unmodified tRNA substrates but was resolved by incubation of substrate tRNAs with Pus10 before *in vitro* methylation studies.

The location of the modification site at the end of the T-loop suggests that TrmY may make contacts with both the D-loop and T-loop to disrupt the interactions between the two tRNA arms to access the target nucleotide. However, TrmY was found to readily modify an isolated T-loop RNA transcript indicating that all structural elements necessary for recognition by TrmY are contained within this region (92). Despite the proximity of the D-loop to the modification site and the extensive interactions between the D- and T-loop, the D-loop does not seem to be essential for substrate recognition by TrmY. However, further kinetic and binding studies are necessary to confirm that the efficiency of methylation of this tRNA fragment is comparable to full-length tRNA.

#### Trm10

Trm10 modifies purines (G and/or A) at position nine of tRNA and is the only SPOUT methyltransferase that modifies a junction nucleotide found in the core of the tRNA (25, 93). Trm10 exhibits a remarkable diversity of target nucleotide specificity between orthologs and paralogs. For example, three paralogs of Trm10 are found in humans: TRMT10A, TRMT10B, and TRMT10C. Each enzyme methylates a unique subset of tRNAs with TRMT10A modifying certain tRNAs containing G9, TRMT10B identified as modifying only one A9-containing tRNA species, and TRMT10C exhibiting bifunctional activity, methylating certain tRNAs containing either G9 or A9 (47, 94, 95, 96). Trm10 from *S. cerevisiae* is the direct homolog of TRMT10A and only modifies certain G9-containing substrates (47, 95). Among archaeal Trm10 orthologs, substrate specificities analogous to those of TRMT10B and TRMT10C have been identified, with Trm10 from *S. acidocaldarius* modifying A9-containing tRNAs and Trm10 from *Thermococcus kodakarensis* modifying G9- and A9-containing tRNAs (22, 26, 47, 95, 96). The molecular basis for these differences in target nucleotide specificity remains poorly understood despite extensive biochemical characterization and availability of multiple structures of different members of the Trm10 family (52, 53, 97, 98).

Trm10 enzymes typically contain both NTD and CTD extensions and function as monomers rather than dimers. However, the length and sequence of the N- and C-terminal extensions can vary drastically even among Trm10 homologs that perform similar modifications (Table 1). Yeast Trm10 enzymes (*S. cerevisiae* and *Schizosaccharomyces pombe*) have similarly sized extended domains with NTD and CTD lengths of around 80 and 20 amino acids, respectively. In contrast, the three human Trm10 paralogs exhibit significant differences in size and sequence of the extended domains, with NTDs of 90, 100, and 140 amino acids for TRMT10A, TRMT10B, and TRMT10C, respectively, and the CTD extensions having around 60, 8, and 20 amino acids for the same enzymes. Archaeal Trm10 from *S. acidocaldarius* has 79 and 46 amino acid extensions on its NTD and CTD, respectively (26). Overall, Trm10 homologs exhibit more diversity in domain length than other SPOUT family members which is likely related to the distinct catalytic activities identified for different Trm10 enzymes. The fact that Trm10 is active as a monomer, unlike other SPOUT methyltransferases, may also be a reason for the greater diversity in domain structure since residues from a second protomer are not available to enable flexible RNA substrate recognition. Structures of multiple Trm10 homologs have revealed that dimerization is likely impeded by the placement of the CTD and the α6 helix which block the typical dimer interface (52, 53). A computational docking model of tRNA to *S. acidocaldarius* Trm10 predicts that the NTD, SPOUT domain, and CTD of Trm10 interact with the entire L-shape structure of the tRNA (53).

A recently solved single-particle cryo-electron microscopy structure of TRMT10C in complex with substrate pre-tRNA reveals more intricate details of how Trm10 interacts with its substrate (98). The structure shows key interactions between TRMT10C and all arms of the tRNA and explains why TRMT10C requires the full tRNA for substrate recognition by identifying both base-specific and nonspecific interactions. In this structure, residues Phe177 and Arg185 in the adapter loops that connect the NTD and SPOUT domain of TRMT10C stack against U35 and C32, respectively, in the tRNA anticodon loop. Arg181 also protrudes into the anticodon loop to interact with the C^2^ carbonyl of U33 in an interaction specific to pyrimidines. The NTD of TRMT10C, which wraps around the tRNA and is lined with positively charged residues, encases the anticodon arm with a connector helix that runs along a groove between the D-loop, anticodon loop, and T-loop. Residue Tyr135 stacks against A47 in the variable region, causing a distortion in the tRNA structure wherein the groove is widened between the D arm and anticodon arm of the tRNA, while the anticodon loop placement is shifted. These insights allow rationalization of the role of the NTD in aiding methyl transfer by TRMT10C.

In the SPOUT domain of TRMT10C, the target nucleotide G9 is flipped out of the tRNA core and stacks with Val313. Interactions between Gln226 and the N^3^ of the primary amine of G9 most likely ensure selectivity of purines at this position. Additionally, Asn350 and Asn348 reach toward the substrate and appear to interact with the carbonyl oxygen of the guanine base (98). TRMT10C also requires dehydrogenase SDR5C1 for activity in the RNase P complex. Further studies and structures will be needed to determine how much relevance this TRMT10C structure, as part of mitochondrial RNase P, has to the specific enzyme–substrate interactions of other Trm10 species that are not part of this complex and act upon different tRNA substrates.

#### RlmH

RlmH is a minimalist SPOUT enzyme which methylates 23S rRNA at Ψ1915 (55, 99) and is thus one of the few SPOUT methyltransferases that requires a prior modification—conversion of uridine to pseudouridine—at its target site to perform methylation (56). Dimerization of RlmH is required to form the active site for rRNA methylation and, as revealed by the structure of *E. coli* RlmH, each protomer appears to be capable of binding to a SAM cofactor. However, the asymmetrical dimerization and proposed positioning of one substrate tRNA per dimer suggest that only one cofactor binding site participates in catalysis. In this arrangement, the SAM binding site of one protomer is oriented to face the proposed RNA binding site of the second protomer where several residues essential for methyl transfer are located (56). Docking models with the bacterial ribosome predict that RlmH makes extensive contacts with both ribosomal subunits, despite its activity targeting only a nucleotide of the large subunit. In these models, RlmH interacts with 16S rRNA nucleotides and ribosomal protein uS12 around the decoding center in the 30S subunit, while contacts between 50S and RlmH are limited to domain IV of the 23S rRNA (56). Although awaiting experimental verification, this model nicely explains the requirement for the full 70S ribosome for RlmH methylation activity, as opposed to an isolated 50S subunit (99, 100).

### Sfm1: A protein-modifying SPOUT methyltransferase

Sfm1 is currently the only known SPOUT methyltransferase that modifies a protein substrate and, along with Trm10, is one of only two SPOUT methyltransferases which are catalytically active as a monomer. Sfm1 catalyzes ω-monomethylation at Arg146 in 40S ribosomal protein uS3 in yeast (20). Arg146 methylation by Sfm1 is predicted to aid import of uS3 to the nucleolus for assembly of the ribosomal small subunit (20). Sfm1 has no detectable methylation activity against isolated uS3 peptides (20), suggesting that the enzyme exploits the full protein tertiary structure of uS3 for specific substrate recognition, similar to the requirement for highly structured targets observed with many RNA-modifying SPOUT superfamily members. Sfm1 contains a SPOUT domain and a CTD extension comprising four β-strands and an α-helix (Table 1) (20). A major difference between Sfm1 and RNA-modifying SPOUT methyltransferases is the negatively charged surface surrounding its active site, including two acidic residues (Glu9 and Glu19) involved in substrate binding, as opposed to the positively charged surfaces implicated in RNA binding for SPOUT RNA methyltransferases (20). Interestingly, despite overall structural similarity to the SPOUT family, the active site of Sfm1 shares several common elements with the structurally unrelated protein arginine methyltransferases PRMT3, PRMT5, and PRMT7 which belong to PRMT classes I, II, and III, respectively. In particular, three catalytically critical Sfm1 residues (Glu9, Trp15, and Glu19) adopt a similar spatial arrangement to analogous essential residues in PRMT3, PRMT5, and PRMT7, but with their organization reversed relative to the target substrates, generating a "mirror image" active site structure between the two types of enzyme (20).

A fourth essential Sfm1 residue (Phe180) that is part of the CTD appears to be involved in positioning the target Arg residue, similar to the role of the extended domains in target RNA recognition for other SPOUT enzymes (20). A Phe180 to alanine mutation renders Sfm1 inactive, while mutation of several negatively charged residues of the CTD also decreases methylation. Thus, Sfm1 activity is dependent upon residues in both its extended domain and the SPOUT domain. The SAM-binding pocket of Sfm1 resembles that of other SPOUT methyltransferases, promoting bound SAM or SAH to assume the signature bent conformation. Based on its crystal structure and complementary gel filtration chromatography and light scattering analyses, Sfm1 is the second known SPOUT methyltransferase that functions as a monomer rather than a dimer. Two α-helices that typically interact to mediate SPOUT dimer formation are unable to do so in Sfm1 due to the steric hindrance between the two protomers, when compared to the dimerization patterns of TrmL and TrmD (20).

Together, these studies reveal that other than possessing the characteristic SPOUT domain in which SAM is bound in its bent conformation, substrate recognition and modification by Sfm1 is quite distinct from other SPOUT methyltransferases with its reversal of typical surface charges, atypical active site organization, and action as a monomeric enzyme. These mechanistic features undoubtably evolved in Sfm1 due to the distinct demands of modifying a protein substrate. Additional studies are needed to determine if other protein-methylating SPOUT methyltransferases exist, which could expand the mechanistic strategies employed by this already diverse collection of enzymes.

## Role of molecular conformational dynamics in substrate recognition and modification

The importance of conformational dynamics is an emerging theme in SPOUT methyltransferase substrate recognition. In addition to the unusual bent SAM conformation enforced by binding the SPOUT domain knot structure, substrate recognition by many SPOUT methyltransferases is a dynamic process that requires specific coordinated conformational changes in the enzyme and/or substrate. This may be the case particularly for enzymes that modify otherwise inaccessible sites in their target RNA substrate.

### Bent SAM conformation

The unique trefoil knot in the SPOUT domain allows SPOUT methyltransferases to enforce a bent conformation in the bound SAM co-substrate in which its methionine moiety is folded toward the adenine base (101). As noted earlier, this bent conformation is necessary for methyl transfer activity and is common among SPOUT methyltransferases regardless of substrate (102). However, there are significant variations in the residues in the active sites of SPOUT methyltransferases that affect how the enzyme binds SAM and the degree to which SAM is bent. Based on observations discussed earlier of the distinct substrate specificities of TrmJ homologs from *E. coli* and *S. acidocaldarius*, there is speculation that the differing bent conformations of SAM may play a role in narrowing or broadening substrate specificity at the target nucleotide for some SPOUT methyltransferases (24). For example, *E. coli* TrmJ binds SAH in a “super-bent” conformation (Fig. 4*C*) stabilized by residue Ser142, and can modify any nucleotide at tRNA position 32. In contrast, *S. acidocaldarius* TrmJ, which has a narrower substrate specificity, has a valine residue (Val139) at the equivalent location which cannot form the same stabilizing interactions. In a Ser142Val variant of *E.coli* TrmJ, the enzyme shifts toward a narrower target site specificity, such that methylation efficiency of U32 is significantly decreased. This finding implies that the superbent SAH conformation creates space to accommodate a larger variety of nucleotides in the active site and that reducing that space by interrupting bonds which stabilize the SAH conformation would narrow specificity. However, additional structural studies on the TrmJ enzymes in complex with substrate RNA are needed to fully clarify the role of the superbent cosubstrate in controlling substrate nucleotide specificity.

For other SPOUT enzymes, there are variations in the degree to which SAM (or SAH) is bent, even within the same crystal structure. For example, the structure of the TrmL–SAH complex shows the dimeric enzyme bound to two SAH molecules, as would be expected for a homodimeric SPOUT methyltransferase. However, while one SAH adopts the signature bent conformation, the other is found in an elongated conformation (Fig. 4*C*) in which it forms an expanded network of interactions with TrmL residues (23). The authors of this study proposed that the altered conformation is due to the presence of a Hepes buffer molecule which resembles the ribose and phosphate of a nucleotide and mimics the bound substrate nucleotide. However, this same conformation is not seen in structures of Nep1 and TrmD bound to substrate RNA (15, 103), and other dimeric SPOUT methyltransferases, such as TsnR, bind both SAM molecules in similar conformations, indicating that this is not necessarily a mechanism shared by all SPOUT methyltransferases (76).

The presence of divalent metal ions may also play a role in ensuring SAM adopts the appropriate bent conformation for some SPOUT enzymes as discussed previously for TrmD. The Mg^2+^ ion causes structural changes that force SAM to adopt its bent conformation and align with active site residues in an optimal orientation for catalysis (84). Why TrmD needs a metal ion to stabilize the bent SAM conformation and for overall enzymatic activity is unclear given that other SPOUT methyltransferase superfamily members apparently do not have this requirement. However, considering that the role of Mg^2+^ in TrmD methyl transfer activity is a relatively recent discovery, future studies may reveal that metal ions play significant mechanistic roles for other SPOUT methyltransferases as well.

### Protein dynamics

Studies to elucidate enzyme conformational changes during substrate recognition demonstrate that for many SPOUT methyltransferases, a certain degree of protein plasticity is necessary for efficient methylation. TrmH is an example of a SPOUT methyltransferase that undergoes an induced-fit process to bind and modify tRNA (28). Substrate recognition for TrmH has been broken down into a two-step process: initial tRNA binding followed by a subsequent induced-fit conformational change in TrmH to accommodate the target nucleotide of the substrate. Kinetic studies show that TrmH binds to nonsubstrate tRNA, including already methylated tRNA, but does not undergo the subsequent conformational changes necessary for methylation. Similarly, other SPOUT methyltransferases, such as Trm10, can bind nonsubstrate tRNA that they are unable to methylate, although the mechanism of substrate discrimination for these enzymes has yet to be elucidated in detail (47). However, recognition mechanisms involving an initial enzyme–substrate docking step and subsequent changes in protein and/or RNA have been demonstrated and proposed for Class I methyltransferases (104, 105) and may be a common feature of RNA modifying enzymes, including the SPOUT family.

The structure of the TrmD–tRNA–sinefungin complex offers a detailed view of conformational changes that take place upon substrate binding for this enzyme (15). Upon tRNA binding, the CTD of one TrmD protomer changes its conformation to snugly contact the tRNA. Additionally, the disordered interdomain linker from the same TrmD protomer forms an α-helix upon tRNA binding. The structure of Nep1 in complex with a model rRNA substrate reveals a similar structural rearrangement at the dimer interface which opens to accommodate the substrate RNA, with the largest conformational changes being observed in the loop regions between the two protomers (88). Other SPOUT methyltransferases that accommodate substrate RNA at the dimer interface are predicted to undergo similar conformational changes that may be revealed through future structural studies.

Trm10 is the only RNA-modifying SPOUT methyltransferase that is active as a monomer and the recent structure of TRMT10C in complex with substrate pre-tRNA (as part of the mitochondrial RNase P complex) provides some insight into structural changes that may occur upon substrate binding (98). The previous Trm10 crystal structure determined without substrate tRNA lacks its NTD, likely due to disorder in this region when not bound to substrate. However, when bound to tRNA, the NTD is α-helical and must exhibit some degree of plasticity to wrap around and bind the tRNA. Superposition of TRMT10C from its complex with pre-tRNA (98) and from the structure of the SPOUT domain bound to SAM (106) show that the catalytic domain remains largely unchanged, aside from local reorganization of a conserved loop (residues 314–319). However, a comparison with the structure of the RNase P complex without SAM suggests that this conformational change occurs upon SAM binding as opposed to with RNA substrate (98). While these findings may help predict the conformational dynamics of other Trm10 enzymes, TRMT10C is unique in that it is part of a larger multisubunit complex which has multiple functions. Other Trm10 enzymes, such as TRMT10A and TRMT10B, may utilize distinct mechanisms of substrate recognition to compensate for the absence of binding partners.

### Protein-induced RNA conformational changes

#### Base flipping and target site reorganization

For DNA and RNA methyltransferases to act on nucleotides that are part of a base pair or take part in stacking, it is often necessary for the target nucleotide base to be rotated ∼180° around its phosphodiester bond so that the base enters the catalytic pocket. Such “base-flipping” is commonly used by methyltransferases, as first observed in the Class I methyltransferase *M.HhaI* DNA C5-methyltransferase complexed with a synthetic DNA complex (1, 107). Structures of TrmD, Nep1, and TRMT10C in complex with their substrate RNAs support the idea that protein binding induces specific conformational changes in the RNA surrounding the target nucleotide (Fig. 5) (15, 88, 98). The crystal structure of the TrmD–tRNA–sinefungin complex reveals that prior to methylation, the G37 base is flipped out from the anticodon loop and protrudes into the catalytic pocket located in the SPOUT domain of one TrmD protomer in the homodimer (15). This flipped conformation is stabilized by Leu160 which stacks on the guanine base and Ser165 which forms a hydrogen bond *via* its side chain OH group with the 2′-OH of G37 (Fig. 5*B*). The N^1^ atom of G37 forms a hydrogen bond with Asp169 which acts as a proton acceptor, with Arg154 also located near the G37 base to stabilize the increased negative charge on the base O^6^ after proton transfer. After G37 is flipped from its original position, nucleotide G36 is stabilized in a *syn* conformation by Asp50 of the second protomer of the TrmD homodimer and is stacked between nearby nucleotides A38 and U35. Additionally, the structure around adjacent nucleotides G36 and A38 opens to make space to allow a TrmD interdomain loop to fold into an α-helix just above the target nucleotide G37. Following these structural reorganization events involving G37 and G36, methylation can occur.

Nep1 uses a similar base-flipping mechanism in the Nep1-rRNA structure in which the target pseudouridine is flipped out from its loop and bound in a pocket in the active site of the Nep1 homodimer (88). The flipped base is stabilized by aspartate and arginine residues in a catalytic pocket at the interface of the two Nep1 protomers (Fig. 5*C*). Finally, in the structure of TRMT10C in the mitochondrial RNase P complex, the G9 target nucleotide in the tRNA core is flipped out of the tRNA fold and buried in the active site to stack against Val313 (98). The base is also stabilized in this flipped conformation by additional neighboring asparagine and glutamine residues (Fig. 5*D*).

Many docking models of other SPOUT methyltransferases, such as RlmH, predict a similar base flipping mechanism to place the target nucleotide in the active site of the enzyme (55). Thus far, however, base flipping has only been demonstrated for base-modifying SPOUT methyltransferases as there are currently no corresponding structural insights for 2′-O-modifying SPOUT RNA methyltransferases. However, a recently determined structure of the mycobacterial Class I 2′-O-modifying rRNA methyltransferase TlyA shows that this enzyme employs base flipping as part of its ribose methylation mechanism, indicating that these specific local conformational changes and base flipping are not exclusive to base-modifying RNA methyltransferases (108). Additional SPOUT methyltransferase–RNA substrate complex structures promise to reveal both common and enzyme-specific mechanistic features of these local conformational changes for both base-modifying SPOUT methyltransferases and 2′-O-modifying methyltransferases.

#### Global changes in RNA structure

Although local rearrangements of RNA structure discussed above appear to be a relatively common feature of modification enzyme mechanisms, conformational changes to the overall substrate RNA structure are comparatively rare. However, TsnR provides one such example of a SPOUT methyltransferase that is proposed to induce this type of large-scale, global conformational change in its 23S rRNA substrate upon binding. Hydroxyl radical probing studies revealed that the backbone of a 58-nucleotide model rRNA substrate is distorted upon TsnR binding due to structural rearrangements in the target loop that are necessary to orient the target nucleotide A1067 at the apex of 23S rRNA Helix 43 into the enzyme’s catalytic site (46). Notably, many of the RNA structural changes observed are distant from the TsnR target site and indicate a more global structural alteration due to TsnR-induced unfolding of the RNA domain’s complex tertiary structure (109). This interpretation was corroborated by ribonuclease RNA structure probing, and the NTD extension appended to the SPOUT domain was identified as the primary driver of RNA structural rearrangements (46). Further, mutation in an internal bulge loop >20 Å away (U1061 to A), which increases the stability of the rRNA tertiary structure, results in a drastic reduction in methyl transfer activity, further supporting the idea that partial unfolding of the global RNA tertiary structure is a key element of specific substrate recognition.

Similar studies using ribonuclease structure probing indicated that upon substrate binding, TrmH induces a conformational change in the tRNA that disrupts tertiary interactions involving the target nucleotide loop. Specifically, for TrmH to gain access to its target nucleotide, enzyme binding may loosen or break D-loop and T-loop interactions resulting in conformational changes to the whole tRNA structure (68). This observation was confirmed using a cross-linking experiment that reduced the flexibility of substrate tRNA and thus hindered binding of TrmH to the D-loop containing the target nucleotide (110). Similar to the stabilization of TsnR’s substrate rRNA through mutation of a distant loop, the stabilization of TrmH’s substrate tRNA through cross-linking resulted in a significant decrease in methylation activity.

Melting assays revealed that Trm56 requires a similar disruption of the interactions between its substrate tRNA D- and T-loops for efficient methylation (70). These studies suggest that Trm56 induces an overall shape transition in substrate tRNA through disruption of key tertiary interactions. These changes shift the tRNA structure from the typical L-shape to an alternative "lambda form" that was first associated with archaeosine modification (111). Critically, this transition is the rate-limiting step for the modification reaction and plays an important role in substrate recognition (70).

Characterization of substrate recognition by other SPOUT methyltransferases hints at similar molecular strategies. For example, TrmJ’s ability to bind to its target nucleotide despite insertion or deletion of nucleotides in the target loop suggests that this enzyme unfolds the target structure upon binding as opposed to binding to a rigid tRNA molecule (24). As more structures become available of SPOUT methyltransferases in complex with their RNA substrate, the precise details of these conformational changes will likely be revealed. For example, Trm10 was predicted to require conformational changes to its substrate tRNA based on the inaccessibility of the target nucleotide in the tRNA core. The structure of the RNase P complex containing TRMT10C revealed a distorted tRNA structure with a 17 Å displacement of the anticodon loop and a considerably larger distance between D-loop and the anticodon loop (98). Significant changes in tRNA conformation are also predicted for other Trm10 enzymes, and these may be distinct from those induced by TRM10C given that they do not employ the additional protein factors of the RNase P complex. We anticipate that the important role of substrate and enzyme conformational plasticity for other SPOUT methyltransferases will continue to be demonstrated as more superfamily members are characterized in molecular detail.

## Conclusions

Despite their shared knotted SAM-binding domain, SPOUT methyltransferases display a remarkable degree of mechanistic diversity, as revealed by many recent advances made through high-resolution structural and biochemical investigations. The new alignment of the SPOUT methyltransferases by their SPOUT domain presented here provides insights into the evolution of the superfamily and could support some inferences for currently uncharacterized members. SPOUT methyltransferases evolved to methylate tRNA and rRNA at the base or ribose and further to modify at least one protein. Throughout evolution of the SPOUT superfamily, NTD/CTD extensions surrounding the SPOUT core have expanded to produce enzymes with or without one or both of these appendages and with significant variation in their lengths and sequences. These distinct domains and their organization confer vastly greater catalytic diversity upon SPOUT enzymes than would likely be achieved with the conserved catalytic SPOUT domain alone. The discovery of the protein-modifying SPOUT methyltransferase Sfm1 suggests that other SPOUT enzymes may exist which act on protein substrates, and future studies may further expand the pool of RNA or protein substrates for enzymes of this superfamily.

Structures of a limited number of SPOUT methyltransferases in complex with their substrates have been critical to begin teasing apart the answers to many questions related to enzyme–substrate interactions, including the basis for substrate selectivity and catalytic mechanism. However, the distinct molecular strategies revealed by these first structures underscore the diversity in structure and mechanism of the SPOUT family enzymes. Because of this diversity, the challenge of generalizing features from one member of the superfamily to another is significant, often even among enzymes in a single organism (such as the case for human Trm10 paralogs). As such, structural determination of many more SPOUT methyltransferases in complex with their substrates will be essential to continue to reveal the full landscape of mechanisms and activities associated with these enzymes.

## Conflict of interest

The authors declare that they have no conflicts of interest with the contents of this article.
